# Influence of cancerostatic perifosine on membrane fluidity of liposomes and different cell lines as measured by electron paramagnetic resonance

**DOI:** 10.3325/cmj.2012.53.558

**Published:** 2012-12

**Authors:** Rok Podlipec, Tilen Koklic, Janez Štrancar, Janez Mravljak, Marjeta Šentjurc

**Affiliations:** 1 Center of excellence NAMASTE, Ljubljana, Slovenia; 2 Jožef Stefan Institute, Ljubljana, Slovenia; 3 Faculty of Pharmacy, University of Ljubljana, Ljubljana, Slovenia

## Abstract

**Aim:**

To test whether membrane fluidity and its changes are important for the sensitivity of cells to the action of perifosine (OPP), a new anticancer drug targeting cell membrane and not DNA.

**Method:**

Influence of OPP on the membrane structure of OPP-resistant MCF7, and OPP-sensitive MT3 breast cancer cell lines, as well as of mouse fibroblasts (L929) cell lines, and model cells (liposomes) was investigated by electron paramagnetic resonance, using spin labeled derivative of OPP (P5) and 5-doxylpalmitoyl methylester (MeFASL(10,3)) as spin probes.

**Results:**

OPP increased membrane fluidity of all cell lines at concentrations higher than 50 µM (on the level of *P* ≤ 0.05, *t* test). In cells, the differences were observed only by P5 and not by MeFASL(10,3). Average order parameter *S_eff_* decreased for about 12% in MCF7 and L929 and only for 8% in OPP-sensitive MT3 cells, showing that there was no correlation between membrane fluidity changes and sensitivity of cells to OPP. The only correlation we found was between OPP sensitivity and the cell growth rate. In liposomes, both spin probes were sensitive to the action of OPP. *S_eff_* decreased with increasing concentration of OPP. For MeFASL(10,3) a significant decrease was observed at 4 mol% OPP, while for P5 it was observed at 8 mol%.

**Conclusion:**

Influence of OPP on plasma membrane fluidity of breast cancer cells is not the determining factor in the sensitivity of cells to OPP.

Perifosine (OPP) belongs to the group of alkylphospholipids (APL), a new class of anticancer agents, targeting directly cell membrane and not DNA. They show a selective apoptotic response in tumor cells, sparing normal cells. The mechanism of APLs action is not yet completely understood. It is known that due to their amphiphilic nature, APLs are easily incorporated into cell membranes in substantial amounts and then spread further into intracellular membrane compartments, where they accumulate and interfere with a wide variety of key enzymes (1,2). At lower, clinically relevant concentrations APLs interfere with phospholipid turnover and lipid-based signal transduction pathways. However, according to recent reviews (3-6), the interaction of APLs differs for different cell lines and tumors. For example, in mouse S49 lymphoma cells they accumulate in detergent-resistant, sphingolipid-, and cholesterol-enriched lipid raft domains and are rapidly internalized by clathrin-independent, raft-mediated endocytosis (7). However, the uptake in human epidrmoide carcinoma cells (strain KB) appears to be raft-independent and is mediated by a yet undefined ATP-dependent lipid transporter (8). Leukemic cells treatment with APLs induces the formation of membrane raft aggregates containing Fas/CD95 death receptor and the adaptor molecule Fas-associated death domain-containing protein, which are critical in the triggering of apoptosis (9). Inhibition of phosphatidylcholine (PC) biosynthesis is another target of APLs that causes cell stress sufficient to trigger apoptosis. In the endoplasmic reticulum of all exponentially growing tumor and normal cells, including leukemic and endothelial cells, APLs inhibit phosphocholine cytidyltransferase (CTP), which catalyzes the rate-limiting step of the de novo PC synthesis (10), which is essential for cell proliferation and is up-regulated in tumor cells.

Early research focusing on the immune stimulating activity of APLs demonstrated that miltefosine (hexadecylphosphatidylcholine) and other lipids of this class are able to activate T-cells and macrophages to express and release chemokines GM-CSF (11), IFgamma (12), and/or nitric oxide (13). They have shown promising results in several clinical studies (14) and among them perifosine (octadecyl(1,1-di-methyl-4-piperidinium-4-yl)phosphate, OPP) and miltefosine seem to be the most promising candidates for breast cancer therapy (15). However, there is a class of breast tumors, mainly those with hormone receptors, that are not sensitive to OPP, while those that lack estrogen receptors seem to be more sensitive (15). The reason for this difference is not yet understood. To clarify this issue, our group has recently synthesized spin labeled derivatives of OPP (16). One of them, P5, was used in our previous work to study the transport of OPP into the OPP sensitive (ER-) MT3 and OPP non-sensitive (ER+) MCF7 breast cancer cells and to measure accumulation of OPP in the cell membrane. From electron paramagnetic resonance (EPR) spectra intensity of P5 immediately after labeling and from the kinetics of nitroxide reduction by oxy-redoxy systems in cells, it was concluded that spin-labeled OPP accumulates better in MT3 than in MCF7 cells (17).

In this study, EPR with spin probes was used to investigate the influence of OPP on the membrane structure of OPP-resistant MCF7 and OPP-sensitive MT3 breast cancer cells. Results were compared to those obtained on fast growing mouse fibroblasts (L929) and model cell membranes (liposomes).

## Materials and methods

**Figure 1:**
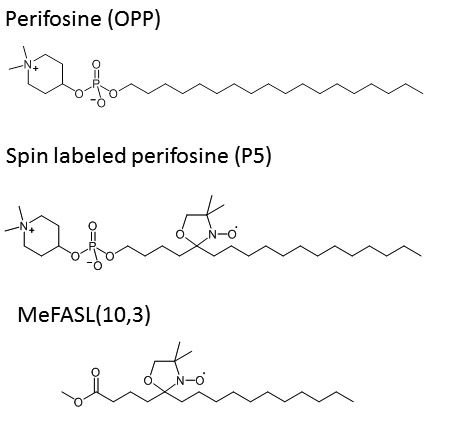
Structural formula of perifosine (OPP) and spin probes: P5 – spin labeled OPP, (MeFASL(10,3) – 5-doxylpalmitoyl methylester; both with the nitroxide group at the 5th C atom (counting from the polar head group).

The spin probes 1,1-dimethylpiperidin-1-IUm-4-yl octadecyl phosphate (Perifosine; ASTA Medica, Frankfurt, Germany), spin labeled perifosine (P5; nitroxide molecule attached on the fifth carbon atom of the alkyl chain counting from the polar head) and 5-doxylpalmitoyl-methylester (MeFASL(10,3)) were synthetized at the Faculty of Pharmacy, University of Ljubljana, Slovenia and used to monitor the membrane properties close to water-lipid interface (Figure 1).

The cell lines studied were experimental human breast cancer estrogen receptor positive (ER+) MCF7, estrogen receptor negative (ER-) MT3 (a generous gift from Dr Zeisig; Experimental Pharmacology Department, Max-DelbruckCentre for Molecular Medicine, Berlin, Germany) and mouse fibroblasts L929 (Educell, Ljubljana, Slovenia).

For liposome preparation, the phospholipids: 1-Palmitoyl-2-oleoyl-phosphatidylcholine (POPC), 1-Palmitoyl-2-oleoyl-phosphatidylethanolamine (POPE), 1-Palmitoyl-2-oleoyl-phosphatidylserine (POPS) (Avanti Polar Lipids, Alabaster, AL, USA) and cholesterol (Serva, Heidelberg, Germany) were used.

### Liposome preparation

Giant liposomes composed of POPC:POPE:POPS:CH in the molar ratio 40:20:10:36 and total lipid concentration of 10 mM were used as a model, which closely mimic the properties of the cell membrane (18). They were prepared according to the procedure described elsewhere (19). In brief, the lipids were dissolved in chloroform and gently introduced into the bottom of 100-mL glass stirring flasks with the total volume of 100 µL. After addition of a few mililiters of phosphate buffer saline (PBS), depending on the chosen final lipid concentration, the mixture was transferred to a rotary evaporator Rotavapor® R-200 (Büchi, New Castle, DE, USA) for 10-minute under 180 mBar. After evaporation of chloroform, a small amount of distilled water was added to compensate for the loss of water during evaporation. The preparation was carried out at the temperature of 57°C, dipping the flask into water bath, which was above phase transition (from gel to ordered liquid phase) and enabled the liposome aggregation.

Giant liposomes were incubated either with MeFASL(10,3) or P5 spin probe and different concentrations of OPP (1, 4, and 8 mol% of total lipids) by a thin lipid film method described in detail elsewhere (20). Mixing of liposome suspension on a thin lipid film using vortex system for 10 minutes enabled the lipids to penetrate into the liposome membrane.

### Cell lines

Human breast cancer cell lines (ER-) MT3 were cultured in RPMI-1640 medium (Gibco, Grand Island, NY, United States) supplemented with 4 μmol/mL L-glutamine (Gibco, Paisley, UK), penicillin-streptomycin with 10 000 units penicillin and 10 mg streptomycin per mL (Sigma, Steinheim, Germany), and heat-inactivated 10% fetal calf serum (PAA, Pasching, Austria), while (ER+) MCF7 and mouse fibroblasts L929 were cultured in DMEM media – GlutaMAX^TM^-I (Gibco) supplemented with fetal calf serum and penicillin- streptomycin in same concentrations. Approximately 10^6^ cells were seeded into a 75 cm^2^ flask at 37°C, 90% humidity, and 5% CO_2_. On the day of the measurement cells were trypsinized at confluence state using Trypsin-EDTA 0.02% in PBS (PAA). On the day of the measurement, the cells were trypsinised in the plateau phase. The cell suspension was centrifuged at cca 400∙g and the pellet (5-15 · 10^6^ cells, dependent on the cell line, cca 20 μL) was resuspended in medium without serum (3 mL). The cell viability was checked by Trypan Blue Stain 0.4% (Gibco) and the number of cells in a sample was calculated after counting the cells using Bürker -Türck's plate.

### Cell labeling and incubation with OPP

Labeling the cells with P5 or MeFASL(10,3) (3 µmol) was performed by a thin lipid film method (21). Briefly, cell suspension (3 mL) in the medium without serum was put into a glass centrifuge tube deposited with a lipid film of spin probe using Rotavapor (Büchi, R-200). Different concentrations of OPP (0, 50, and 150 µM) were deposited on the wall of the tube together with the spin probe. The cell suspension was put on a vortex system for 10 minutes in order to detach spin labels and OPP and to distribute them into the cell membranes due to their amphiphilic nature. Labeled cell suspension with or without OPP was then centrifuged at cca 320 · g for 2 minutes and after removal of supernatant the cell pellet was transferred into a 1 mm glass capillary (Euroglass, Ljubljana, Slovenia) and immediately put in an X-band EPR spectrometer (ELEXSYS-II 500 Bruker, Bremen, Germany).

### Electron paramagnetic resonance

EPR spectra were acquired at 20°C (center field 332 mT; sweep width 10 mT; modulation amplitude 0.15 mT; modulation frequency 100 kHz, and microwave power 20 mW).

**Figure 2:**
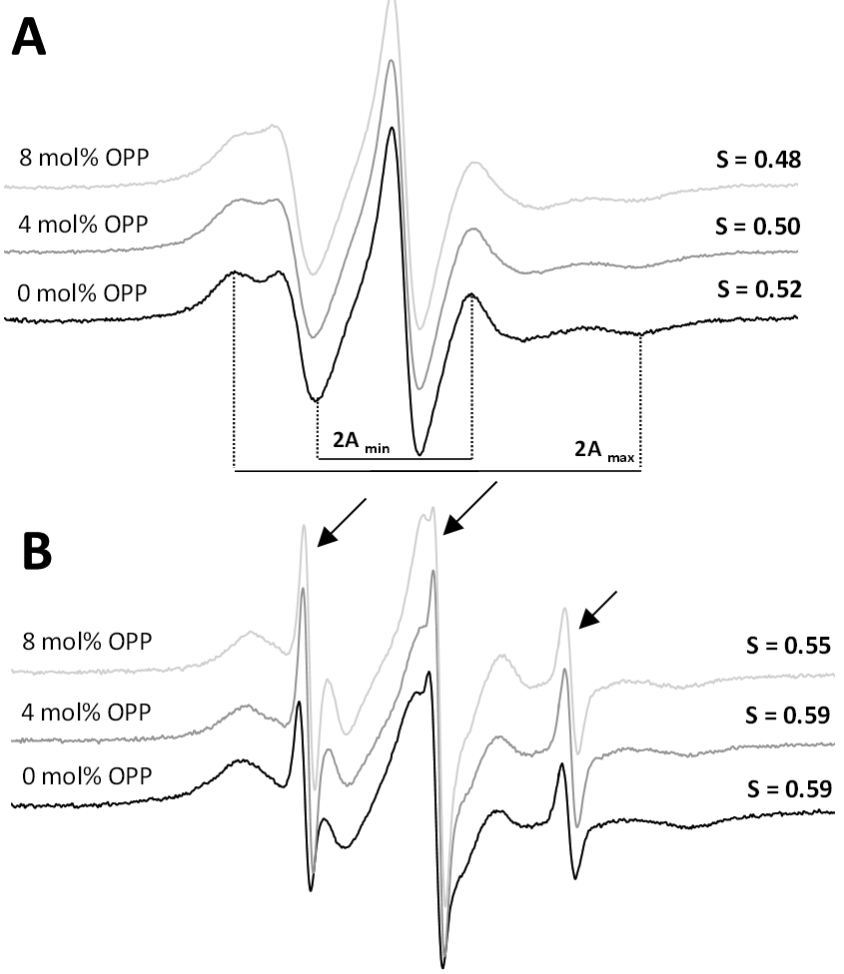
Electron paramagnetic resonance (EPR) spectra of (**A**) MeFASL(10,3) and (**B**) P5 in the membranes of giant liposomes composed of POPC:POPE:POPS:CH (40:20:10:36) in the presence of different concentrations of perifosine (OPP); the isotropic part is indicated by arrows. Effective order parameters *S_eff_* are also given.

From the spectra, maximal and minimal hyperfine splitting (2A_max_ and 2A_min_) were measured as shown in Figure 2 and an effective order parameter S_eff_ was calculated:

*S_eff_ = (A_max_ – A_min_) a_N_/(A_zz_ – (A_xx_ + A_yy_)/2) a_N_’* (1)

a_N_’ = (A_max_ + 2A_min_)/3,

where *A_xx_*, *A_yy_*, *A_zz_* are eigen values of hyperfine coupling tensor of the spin label in monocrystal – for MeFASL(10,3): *A_xx_* = 0.63 mT; *A_yy_* = 0.58 mT; and A_zz_ = 3.36 mT (22). *S_eff_* gives a rough estimation of the average ordering of the acyl chains in the membrane and the changes caused by the interaction with OPP, taking into account that the motion of nitroxide group is influenced by the motion of the surrounding acyl chains.

### Computer simulation of EPR spectra

EPR data measured directly from the spectra give information on the average membrane fluidity in the vicinity of the probes without distinguishing whether the membrane is heterogeneously composed of regions with different fluidity characteristics (inner and outer leaflet, close to the polar head groups, in the middle of the membrane, lateral heterogeneity, etc). For more precise description of membrane characteristics, a computer simulation of EPR spectra is necessary, which takes into account that the experimental EPR spectrum is a superposition of several spectral components reflecting different modes of restricted rotational motion of spin probes in different environments of the membrane (23,24). Each spectral component is described with a set of spectral parameters: order parameter (*S*), rotational correlation time *(τ_c_*), the line-width broadening (*W*), and the polarity correction of the magnetic tensors *g* and *A* (*p_g_* and *p_A_*, respectively). Order parameter describes the orientational order of the phospholipids’ acyl chains in the membrane regions and varies from *S* = 1 for perfectly ordered membrane to *S* = 0 for isotropic distribution; rotational correlation time (*τ_c_*) describes the rate of nitroxide group motion, which is influenced by the acyl chain motion of the surrounding lipids; polarity correction factors *p_g_* and *p_A_* arise from the polarity of the spin probe nitroxide group environment; and the line-width broadening (*W*) appears due to the paramagnetic impurities, spin-spin interactions, unresolved hydrogen super-hyperfine interactions, etc. Besides, a fraction (*weight*) of each spectral component has to be determined, which describes the relative amount of the spin probes with particular motional mode. Since partition of MeFASL(10,3) was found to be approximately equal between different membrane regions of phospholipid/cholesterol vesicles (25), we assumed that the same was valid also for liposomes and cells used in this study. It should be stressed that the lateral motion of the spin probe within the membrane is slow on the EPR time scale (26). Therefore, each spectral component describes only the properties of the spin probe nearest surroundings on the nanometer scale and represents the sum of all membrane regions with the same properties.

To obtain the best fit of the calculated EPR spectrum to the experimental one, the multi-run hybrid evolutionary optimization algorithm was used (23), together with a newly developed GHOST condensation procedure that enables to determine the number of different membrane regions (24) and is implemented in the software package EPRSIM (*http://www.ijs.si/ijs/dept/epr*/).

### Determination of critical micellar concentration

Critical micellar concentration (CMC) of OPP and P5 was determined with Krüss processor tensiometer, as described in more details elsewhere (16). CMC value for OPP was between 2.6 and 3.4 µM and was in the same range as was published for miltefosin (27). For P5, CMC was 4.22 µM, in accordance with previously published results (16).

## Results

### Cell growth and viability in the presence of OPP

Cell viability determined using Trypan Blue Stain depended on the OPP concentration added (Table 1). At 50 µM OPP concentration, a minimal cytotoxic effect was observed on MT3, while no effect was found on MCF7 and L929 cell lines. At 150 µM, OPP cytotoxic effect increased rapidly on L929 cell lines, where only a small fraction of cells survived (10% to 30%), while on cancer cell lines cytotoxic effect was much lower and was slightly higher for MT3 than for MCF7.

**Table 1:** Viability of MCF7, MT3, and L929 cell lines (%) after addition of different concentrations of perifosine (OPP) in 1 mL cell suspension and the rate of cell growth in cell culture flasks as the number of days needed to grow to confluent state (the same number of cells was seeded into each culture flask).

Cell line	OPP (µM)			Cell growth (days/confluence)
	0	50	150	
MCF7	70-90	70-90	60-80	3
MT3	70-90	60-80	50-70	2
L929	70-90	70-90	10-30	1

Before measuring cell viability and performing further EPR studies, the rate of cell growth was evaluated for all cell lines (Table 1). After harvesting (trypsination) of cells in the subconfluent state, cells were further seeded into 2 cell culture flasks (half of the cells in each flask) and the time needed to grow to confluent state was measured. L929 cell growth and differentiation was fastest while that of MCF7 was slowest, indicating that cell viability in the presence of OPP correlated with the rate of cell growth.

### EPR measurements

*Model cells – liposomes.* EPR spectra of MeFASL(10,3) and P5 in giant liposomes changed with different concentrations of OPP (Figure 2). *S_eff_* decreased with the increasing concentration of OPP, indicating that OPP was incorporated into the liposome membrane and that it increased its average fluidity. MeFASL(10,3) spin probe was slightly more sensitive to the perturbation of the liposome membrane by OPP than P5. With MeFASL(10,3) the difference in spectra line-shape was detected already at 4 mol% of OPP, while 8 mol% of OPP was needed to see any spectral change with P5. Distribution of MeFASL(10,3) and P5 in different membrane local environments was well visible from the differences in the EPR spectra line-shape between the two probes (Figure 2, A and B), which showed less ordered environment of nitroxide group of MeFASL(10,3) than of P5. In the spectra of P5 also the isotropic part was visible, due to the presence of free P5 in the solution (in Figure 2.B indicated by arrows). However, there was no isotropic part in MeFASL(10,3) spectra due to the lipophilic character of this molecule.

More information about the structural changes in the liposome membrane was obtained by computer simulation of the EPR spectra. To get a good fit with experimental spectra it was necessary to take into account three spin probe environments with different modes of spin probe motion, indicating three types of regions in the membrane with different fluidity characteristics (R1, R2, and R3 in Table 2). The fraction of spin probes (*weight*) was highest in the most ordered regions (R1), indicating that these regions occupy the largest area of the membrane. OPP decreased order parameter *S* of R1 regions as detected with both spin probes, indicating higher fluidity of the most ordered domains (Table 2). The order parameter of regions R2 increased and as a consequence the spin probe P5 detected merging of R2 and R1 regions. The order parameter of the less ordered regions R3 remained in the range of experimental errors. In these regions, OPP increased polarity correction factor *p_A_* as detected by MeFASL(10,3) spin probe (Table 2, MeFASL(10,3), region R3). In the regions with the intermediate order parameter (R2), OPP decreased the rotational correlation time (Table 2, MeFASL(10,3), region R2).

**Table 2:** Electron paramagnetic resonance (EPR) parameters of the best fits of the calculated spectra obtained by EPRSIMC program with the experimental spectra of MeFASL(10,3) and P5 in the membrane of giant liposomes composed of POPC:POPE:POPS:CH (40:20:10:36) without perifosine (OPP) and in the presence of 8 mol% OPP; R1, R2, R3 denote three types of regions with the same motional characteristics (R1 for the most ordered region and R3 for less ordered region); *weight* – proportion of spin probes with the same motional characteristics; *S* – order parameter, *τ_c_* – rotational correlation time; and *p_A_* – polarity correction factor of hyperfine splitting constant

Region	OPP (%)	*Weight* (%)	*S*	*τ_c_* (ns)	*p_A_*	
**MeFASL(10,3)**						
R1	0	74	0.62 ± 0.04	0.17 ± 0.06		0.98 ± 0.01
	8	75	0.59 ± 0.03	0.24 ± 0.12		0.99 ± 0.01
R2	0	17	0.29 ± 0.01	3.01 ± 0.36		1.05 ± 0.01
	8	14	0.37 ± 0.02	**1.2**4 ± 0.29in		0.98 ± 0.01
R3	0	9	0.21 ± 0.01	0.64 ± 0.21		0.84 ± 0.02
	8	11	0.19 ± 0.01	1.66 ± 0.14		0.95 ± 0.01
**P5**						
R1	0	66	0.70 ± 0.02	0.13 ± 0.03		1.00 ± 0.01
	8	78	0.63 ± 0.02	0.29 ± 0.10		0.99 ± 0.01
R2	0	24	0.59 ± 0.02	0.14 ± 0.05		1.00 ± 0.01
	8	/	/	/		/
R3	0	5	0.42 ± 0.01	0.90 ± 0.20		1.01 ± 0.02
	8	11	0.42 ± 0.01	0.74 ± 0.13		0.99 ± 0.01

**Figure 3:**
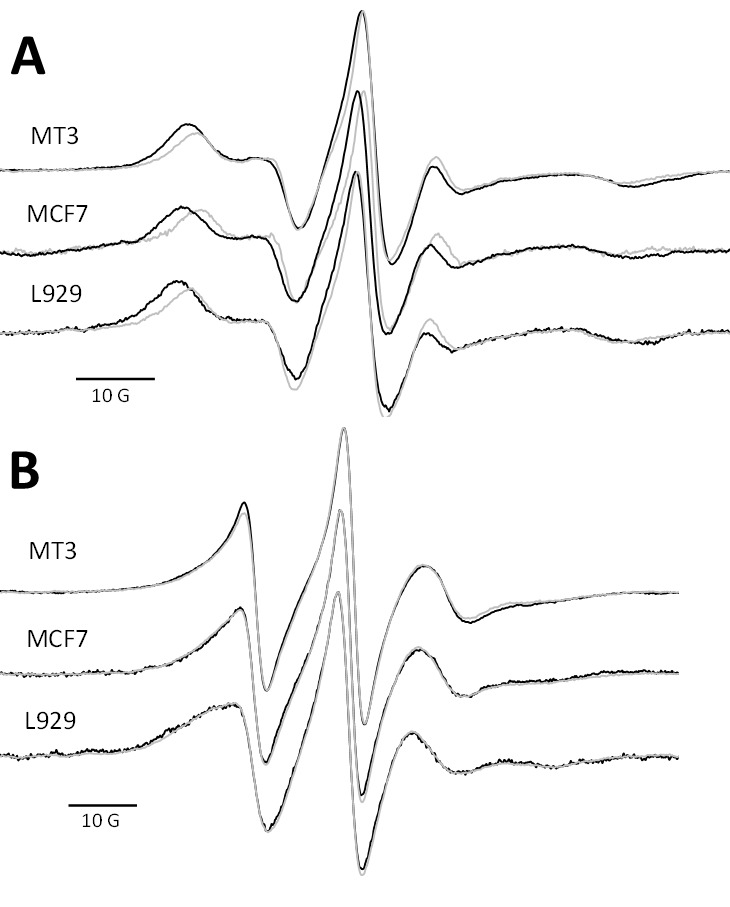
Electron paramagnetic resonance (EPR) spectra of (**A**) P5 and (**B**) MeFASL(10,3) in the membranes of OPP sensitive MT3, perifosine (OPP) resistant MCF7 breast cancer cell lines, and L929 cell lines. EPR spectra were measured at room temperature in the presence (gray) and absence of OPP (black).

*Cells.* EPR spectra of MeFASL(10,3) and P5 in the membrane of different cell lines showed the spectra typical for restricted spin probe motion (Figure 3). Similarly as in liposomes, the line shapes of EPR spectra of the two spin probes were different, indicating different position within the membrane. MeFASL(10,3) monitored more fluid environment than P5. Effective order parameters (*S_eff_*) of P5 were calculated directly from the spectra according to the equation 1 (Table 3). At room temperature, MeFASL(10,3) did not detect any significant changes in the presence of OPP (50 µM and 150 µM), while for P5 significant differences were observed after addition of 150 µM OPP for all three cell lines. In normal fibroblasts L929, the changes were visible already at 50 µM OPP concentration, indicating that these fast growing cell lines were most sensitive to OPP, as already suggested by measuring the lowest viability after OPP admission (Table 1). However, comparing the results in Table 1 and Table 3, it was evident that membrane fluidity changes were not directly correlated with cytotoxic effect of OPP. For L929 cells, *S_eff_* decreased already at 50µM OPP, although at this concentration viability of cells was not yet diminished. For the two breast cancer cell lines, no significant difference in *S_eff_* was observed at 50 µM OPP, while at 150 µM OPP the changes were more pronounced for OPP- resistant MCF7 cells. Also, there was no relation between *S_eff_* of the cell lines investigated and their sensitivity to OPP. The most sensitive L929 had the highest effective order parameter (*S_eff_ =* 0.72 ± 0.01), while the opposite effect was found for breast cancer cell lines; for OPP-sensitive MT3 *S_eff_ =* 0.68 ± 0.01 and for OPP-resistant MCF7 *S_eff_ =* 0.70 ± 0.01.

**Table 3:** Effective order parameters *S_eff_* of P5 in the membrane of perifosine (OPP)-resistant MCF7, OPP-sensitive MT3 breast cancer cell lines, and normal mouse fibroblasts L929 at room temperature for different concentrations of OPP

Cell line	OPP (µM)		
	0	50	150
MCF7	0.70 ± 0.01	0.70 ± 0.01	0.61 ± 0.01
MT3	0.68 ± 0.01	0.67 ± 0.01	0.62 ± 0.01
L929	0.72 ± 0.01	0.67 ± 0.01	0.64 ± 0.01

Similarly as in the spectral analysis of liposomes, in cell membranes good fit of experimental spectra was obtained with three spectral components. The main contribution to the decrease in the effective order parameter, measured directly from the spectra, in the presence of OPP was the decrease in the fraction of the region with the highest order parameter (R1) and the increase in the fraction of the region with intermediate order parameter (R2) (Table 4). The most significant differences were observed in L929 cells, where the fraction of region R2 increased from 7% to 42% at 150µM OPP. The changes were less pronounced in cancer cells, but seemed to be slightly larger for OPP-resistant MCF7 than for OPP-sensitive MT3 cells. Contrary to the noticeable changes in the distribution of the regions with different order parameter caused by addition of OPP, there were only minimal changes in the EPR parameters. The only significant difference was observed in the decreased polarity correction factor *p_A_* in the presence of OPP in the two most ordered regions for all cell lines, indicating that OPP decreases permeability of the cell membrane for polar molecules.

**Table 4:** Electron paramagnetic resonance (EPR) parameters of the best fits of the calculated spectra obtained by EPRSIMC program with the experimental spectra of P5 in the membrane of MCF7, MT3, and L929 cell lines for different concentrations of OPP measured at room temperature; weight – proportion of spin probes with the same motional characteristics; *S* – order parameter; *τ_c_* – rotational correlation time; and *p_A_* – polarity correction factor of hyperfine splitting constant

Region	OPP (µM)	*Weight* (%)	*S*	*τ_c_* (ns)	*p_A_*
MCF7					
R1	0	84	0.77 ± 0.02	0.12 ± 0.02	1.02 ± 0.01
	50	81	0.81 ± 0.03	0.11 ± 0.02	1.02 ± 0.01
	150	62	0.79 ± 0.02	0.13 ± 0.03	1.00 ± 0.01
R2	0	13	0.60 ± 0.01	0.38 ± 0.08	1.04 ± 0.01
	50	14	0.63 ± 0.02	0.30 ± 0.11	1.03 ± 0.01
	150	34	0.60 ± 0.02	0.39 ± 0.08	1.00 ± 0.01
R3	0	4	0.27 ± 0.01	0.39 ± 0.10	1.20 ± 0.01
	50	5	0.28 ± 0.01	0.42 ± 0.09	1.19 ± 0.01
	150	4	0.23 ± 0.01	0.33 ± 0.08	1.18 ± 0.02
MT3					
R1	0	89	0.73 ± 0.01	0.12 ± 0.02	1.02 ± 0.01
	50	84	0.76 ± 0.02	0.12 ± 0.02	1.02 ± 0.01
	150	70	0.76 ± 0.02	0.13 ± 0.02	1.00 ± 0.01
R2	0	6	0.59 ± 0.01	0.44 ± 0.09	1.04 ± 0.01
	50	12	0.62 ± 0.01	0.33 ± 0.12	1.04 ± 0.01
	150	26	0.62 ± 0.02	0.30 ± 0.06	1.01 ± 0.01
R3	0	5	0.29 ± 0.01	0.46 ± 0.10	1.20 ± 0.01
	50	4	0.30 ± 0.01	0.47 ± 0.11	1.20 ± 0.01
	150	4	0.25 ± 0.01	0.35 ± 0.11	1.20 ± 0.01
L929					
R1	0	87	0.78 ± 0.01	0.13 ± 0.04	1.03 ± 0.01
	50	75	0.79 ± 0.02	0.12 ± 0.03	1.02 ± 0.01
	150	53	0.80 ± 0.02	0.11 ± 0.02	1.01 ± 0.01
R2	0	7	0.69 ± 0.02	0.34 ± 0.09	1.03 ± 0.01
	50	19	0.68 ± 0.02	0.34 ± 0.09	1.02 ± 0.01
	150	42	0.66 ± 0.02	0.25 ± 0.07	1.00 ± 0.01
R3	0	6	0.34 ± 0.01	0.57 ± 0.12	1.19 ± 0.01
	50	6	0.31 ± 0.01	0.44 ± 0.08	1.20 ± 0.01
	0	5	0.27 ± 0.01	0.43 ± 0.11	1.20 ± 0.01

## Discussion

The results showed that our hypothesis that sensitivity of breast cancer cells to OPP was connected with cell membrane fluidity was not confirmed, but a possible correlation between the rate of cell growth and sensitivity of cells to OPP was indicated.

In first part of our study we determined the influence of OPP on the membranes of giant liposomes composed of the most common cell membrane lipids. They were used as a model for cell membrane (18) but without rafts, receptors, or enzymes. To the best of our knowledge, there are no published data about the influence of OPP on model cell membranes. There are some available findings for some other APLs, like miltefosine (HePC) (28) and edelfosine (ET-18-OCH_3_) and its analogues (29,30), showing membrane fluidity increase in the presence of APLs. This is in accordance with our experiments. We found that OPP decreased an average ordering of the membrane when the ratio of OPP to lipids was more than 4 mol%. An increase in membrane fluidity was observed with both spin probes, indicating a uniform distribution of OPP in model membranes. Computer simulation of EPR spectra showed that increased fluidity in the presence of OPP was a consequence of a decrease in order parameter of the largest and most ordered membrane regions. Furthermore, OPP caused an increase in the polarity correction factor (*p_A_*) of less ordered regions as detected by MeFASL(3,10), indicating that it promoted partitioning of water molecules into these regions. In the regions with the intermediate order parameter (R2), OPP caused a decrease in the rotational correlation time (MeFASL(10,3), region R2). Comparable changes were measured previously in liposomes of similar composition at concentration of OPP above 30 mol% of membrane constituents (unpublished results).

The influence of OPP on the membrane properties was more pronouncedly detected using MeFASL(10,3) than P5 as a spin probes. This indicates greater influence of OPP in the part of the membrane where nitroxide group of MeFASL(10,3) was distributed. MeFASL(10,3) is highly lipophilic, with partition coefficient between membrane and solution log *P* = 5.12 (31). It distributes inside the membrane and can exhibit some translational motions due to the non-polar character of the methyl group. Therefore, the position of its nitroxide group within the membrane is not well defined. On the other hand, P5 has the positive charge on the polar head, which fixes its position in the polar head region of the membrane phospholipids, which is reflected in more restricted motion of nitroxide groups of P5.

The effect of OPP on model membranes is expected as it is known that APLs insert progressively from the aqueous medium into lipid monolayers as monomers below the critical micellar concentration (CMC), and as the combination of monomers and micelles above the CMC (27). Like other APLs, OPP is an amphiphilic molecule with solubility of 10 mg/mL and CMC in the range of 2.6- 3.4 µM. It is distributed between aqueous medium and lipid layers and therefore it lowers the mol% concentration of OPP in the membranes. As P5 is a spin labeled analogue of OPP, it was expected to be distributed between the membrane and solution similarly as OPP. It is soluble in water and forms micelles above CMC = 4.22 µM (16). Distribution of P5 between aqueous medium and lipid layers is reflected in the isotropic part of its spectra in liposomes (Figure 2B).

In the second part of our investigation, we determined the influence of OPP on two breast cancer cell lines and mouse fibroblasts. The influence of OPP on cell membrane fluidity was less pronounced than in model membranes (higher amount of OPP was needed to observe the difference). This indicated that different metabolic processes in the cells accelerated the transport of OPP into the cell interior or prevented OPP uptake by the cells.

It is interesting to note that in contrast to the significant influence of OPP on cell membrane fluidity observed with spin probe P5, no influence was observed with MeFASL(10,3). The possible reason could be different locations of MeFASL(10,3) and P5 in the membrane, which is evident from the line-shape of the EPR spectra. In the model membranes, MeFASL(10,3) is distributed equally in all membrane regions and is rapidly equilibrated between both layers of the membrane (25). We suppose that the same is also valid for cell membranes. On the other hand, the spin probe P5 is specially designed to mimic the structure of OPP and differs from OPP only by additional nitroxide group at the 5th C atom. It is possible that P5 did not distribute uniformly in the membranes, but interacted with them in similar way as OPP and other alkylphospholipids, which interact with different cell lines in different manner (7-10). For example, in mouse S49 lymphoma cells, APLs accumulate in lipid raft domains and are rapidly internalized by raft-mediated endocytosis (7), while in some other cell lines, like in KB carcinoma cells, their uptake was mediated by an ATP-dependent lipid transporter (8). Besides, the positive charge on the polar head of P5 prevented fast transport of P5 from the outer cell layer into the inner layer and into the cell (32). Therefore, we suppose that P5 was not equally distributed between different membrane regions but was preferentially located in the region targeted by OPP.

When we compared viability and membrane fluidity of cells in the presence of OPP, the most sensitive to OPP was the mouse fibroblast cell line L929, which is also the most rapidly growing. This is in accordance with the studies that measured the correlation between the degree of proliferative activity of cells and the degree of apoptosis (10). Not only cancer cells but also normal cells are sensitive to APLs as long as they are in the proliferative state.

Our viability study of breast cancer cell lines, which showed lower cytotoxicity of OPP for MCF7 cells as for MT3 cells confirmed that MT3 were more sensitive to OPP than MCF7 (15,33). Furthermore, our previous results also showed better accumulation of OPP into MT3 cell membranes (17). Irrespective of that, the membrane fluidity of MT3 was slightly less influenced by OPP than it was the case with MCF7 cells. Besides, the effect of OPP on cell viability (IC_50_) was observed at lower concentrations (below 20 µM) (33,34) than was the concentration needed to affect the membrane fluidity (above 50 µM). Therefore, we suppose that the changes in membrane fluidity caused by OPP are not directly correlated with the sensitivity of cells to OPP. The only correlation observed was between OPP sensitivity and the cell growth rate.

According to our knowledge, there are only few data about the influence of APLs on cell membrane fluidity showing similar membrane fluidity increase as observed in this study, but using other APLs and other cell lines (28-30). Higher fluidity increase in presence of miltefosine was found for multidrug sensitive mouse fibroblasts in comparison to multidrug resistant ones (28). In our study, such correlation between the cells sensitive to OPP and the resistant ones was not observed, indicating that the transport mechanisms acting in multidrug resistant cells are different from those in breast cancer cell lines experiencing different sensitivity to APLs. In this study, only three cell lines with different growing rates and different sensitivity to OPP were compared, which is not enough to draw a conclusion about the possible correlation between the growing rate and sensitivity to OPP.

So far we proved that plasma membrane fluidity of cells and fluidity changes caused by OPP were not the determining factors in sensitivity of cells to OPP. In the future, our investigations should be extended to more cell lines with different growing rates and sensitivity to OPP. Beside membrane fluidity changes, the uptake and accumulation of OPP by different cell lines should be followed and possible transport mechanisms should be further investigated. Only an extensive investigation of cell-OPP interaction using different methods and approaches will make this promising anticancer drug more reliable for cancer treatment.
